# Letter to the Editor Regarding: “Padilha VH, et al. Lumbopelvic Muscle Mobility and Resistance and Their Association with Musculoskeletal Pain in Ballet Dancers. Rev Bras Ortop (São Paulo). 2023;58(3):410–416”

**DOI:** 10.1055/s-0044-1787771

**Published:** 2024-07-15

**Authors:** André Pontes-Silva

**Affiliations:** 1 Programa de Pós-Graduação em Fisioterapia, Departamento de Fisioterapia, Universidade Federal de São Carlos, São Carlos, SP, Brasil

In a study recently published in *Revista Brasileira de Ortopedia* , Padilha et al. 1 analyzed ankle and lumbopelvic muscle mobility, resistance, and factors associated with musculoskeletal pain in young ballet dancers. The main complaints reported by the ballet dancers were low back pain and lower extremity pain. Those with low back pain presented significantly lower lumbar mobility ( *p* = 0.05) and lower ankle mobility bilaterally ( *p* ≤ 0.05). 1 The authors are to be congratulated for the article; however, an important gap regarding evaluation needs to be discussed so that we can move forward scientifically.

Padilha et al. 1 used the Leg Lateral Reach Test (LLRT) 2 to assess the thoraco-lumbo-pelvic rotation range of dancers. They placed the participants in the supine position with their arms at their sides to perform hip flexion with unilateral knee extension (only the side being evaluated) and thoraco-lumbo-pelvic rotation range with the shoulders maintaining contact with the floor. 1 The goal of the test is to reach a previously marked measuring tape perpendicular to the popliteal fossa on the side that is contralateral to the one being tested (right and left sides). 2

Although the LLRT is reliable (intraclass correlation coefficient, ICC ≥ 0.889) and inexpensive, 3 it is a new and extremely-limited test. It was developed by Kim et al. 2 (2017) for healthy subjects and adapted by Pontes-Silva et al. 3 (2021) for patients with chronic low back pain. To date, only these three studies 1 2 3 have used the LLRT, and none of them has established a cut-off point to diagnose adequate/inadequate thoraco-lumbo-pelvic rotation range, making it just another test whose evaluation generates data that cannot define a prognosis. 1 2 3

The study by Padilha et al., 1 for example, showed that patients presented rotational mobility to the right with a mean distance of 69.8(± 5.47) cm and, to the left, of 80.7(± 31.56) cm. However, the lack of a cutoff point to diagnose thoraco-lumbo-pelvic rotation hypomobility using the LLRT makes it impossible to state that these values (69.8 cm or 80.7 cm) indicate adequate or inadequate thoraco-lumbo-pelvic rotation range.

Therefore, further research should be conducted to assess whether the LLRT has a cutoff point in healthy individuals to diagnose thoraco-lumbo-pelvic rotation hypomobility in patients with low back pain. Finally, two questions remain unanswered: what is the cutoff point to diagnose thoraco-lumbo-pelvic rotation hypomobility using the LLRT? And what is the variation in the sensitivity and specificity of the LLRT using a receiver operating characteristic (ROC) curve?
