# Supplementary material for: Letter to the Editor Regarding: “Padilha VH, et al. Lumbopelvic Muscle Mobility and Resistance and Their Association with Musculoskeletal Pain in Ballet Dancers. Rev Bras Ortop (São Paulo). 2023;58(3):410–416”
Source: Rev Bras Ortop (Sao Paulo). 2024 Jul 15;59(5):e806–8. doi: 10.1055/s-0044-1787771 (PMC11624939; doi:10.1055/s-0044-1787771)
Supplement: Related texts

# Research article: Carta ao editor referente a “Padilha VH, et al. Mobilidade e resistência muscular lombopélvica e associação com dor musculoesquelética em bailarinas. Rev Bras Ortop (São Paulo). 2023;58(3):410–416”

Em um estudo recentemente publicado na *Revista Brasileira de Ortopedia* , Padilha et al. 1 analisaram a mobilidade, a resistência e os fatores musculares do tornozelo e da musculatura lombopélvica associados à dor musculoesquelética em jovens bailarinas. As principais queixas relatadas pelas bailarinas foram lombalgia e dores nos membros inferiores. Aquelas com dor lombar apresentaram mobilidade lombar significativamente menor ( *p* = 0,05) e menor mobilidade bilateral do tornozelo ( *p* ≤ 0,05). 1 Congratulo os autores pelo artigo, mas precisamos discutir uma lacuna importante quanto à avaliação precisa para que possamos avançar cientificamente.

Padilha et al. 1 utilizaram o *Leg Lateral Reach Test (* LLRT) 2 para avaliar a amplitude de rotação toracolombopélvica de bailarinas. Os autores posicionaram as participantes em decúbito dorsal com os braços ao lado do corpo para realizar a flexão do quadril com extensão unilateral do joelho (apenas do lado avaliado) e a amplitude de rotação toracolombopélvica sem perder o contato dos ombros com o chão. 1 O objetivo do teste é alcançar uma fita métrica previamente marcada, perpendicular à fossa poplítea, no lado contralateral ao testado (lados direito e esquerdo). 2

Embora o LLRT seja confiável (coeficiente de correlação intraclasse [CCI] ≥ 0,889) e barato, 3 é um teste novo e extremamente limitado. O LLRT foi desenvolvido por Kim et al. 2 (2017) para indivíduos saudáveis e adaptado por Pontes-Silva et al. 3 (2021) para pacientes com dor lombar crônica. Até o momento, somente esses três estudos 1 2 3 usaram o LLRT, e nenhum deles estabeleceu um ponto de corte para o diagnóstico da amplitude de rotação toracolombopélvica adequada/inadequada. Assim, o LLRT é apenas mais um teste cuja avaliação gera dados que não conseguem definir um prognóstico. 1 2 3

O estudo de Padilha et al., 1 por exemplo, mostrou que os pacientes apresentavam mobilidade rotacional para a direita com distância média de 69,8(± 5,47) cm e, para a esquerda, de 80,7 ( ± 31,56) cm. Entretanto, a falta de um ponto de corte para o diagnóstico de hipomobilidade de rotação toracolombopélvica pelo LLRT impossibilita afirmar que esses valores (69,8 cm ou 80,7 cm) indicam amplitude de rotação toracolombopélvica adequada ou inadequada.

Portanto, novas pesquisas devem ser realizadas para verificar se o LLRT tem um ponto de corte em indivíduos saudáveis para o diagnóstico de hipomobilidade de rotação toracolombopélvica em pacientes com dor lombar. Por fim, duas questões continuam sem resposta: qual o ponto de corte para diagnóstico de hipomobilidade de rotação toracolombopélvica pelo LLRT? E qual é a variação na sensibilidade e na especificidade do LLRT usando uma curva de característica de operação do receptor (ROC)?
